# Tunable nanoplasmonic sensor based on the asymmetric degree of Fano resonance in MDM waveguide

**DOI:** 10.1038/srep22428

**Published:** 2016-03-02

**Authors:** Shiping Zhan, Yongyi Peng, Zhihui He, Boxun Li, Zhiquan Chen, Hui Xu, Hongjian Li

**Affiliations:** 1 College of Physics and Electronics, Central South University, Changsha 410083, China; 2 College of Physics and Electronics Science, Hunan University of Science and Technology, Xiangtan, 411201, China

## Abstract

We first report a simple nanoplasmonic sensor for both universal and slow-light sensing in a Fano resonance-based waveguide system. A theoretical model based on the coupling of resonant modes is provided for the inside physics mechanism, which is supported by the numerical FDTD results. The revealed evolution of the sensing property shows that the Fano asymmetric factor *p* plays an important role in adjusting the FOM of sensor, and a maximum of ~4800 is obtained when *p* = 1. Finally, the slow-light sensing in such nanoplasmonic sensor is also investigated. It is found that the contradiction between the sensing width with slow-light (SWS) and the relevant sensitivity can be resolved by tuning the Fano asymmetric factor *p* and the quality factor of the superradiant mode. The presented theoretical model and the pronounced features of this simple nanoplasmonic sensor, such as the tunable sensing and convenient integration, have significant applications in integrated plasmonic devices.

## Introduction

Since the surface plasmons (SPs) can overcome the diffraction limit and manipulate light in nanoscale domain, they provide possibility for nanodevices with extraordinary properties, high miniaturization and integration^1^. Plasmonic sensor is a kind of nanoscale device by utilizing the extreme sensitivity of SPs to the surrounding dielectric environment^2^. The Fano resonance results from the coupling effect between a wide continent state and a narrow distinct state, leading to the sharp and asymmetric spectral line shape, and small perturbations can induce dramatic intensity variation or spectrum shifts^3^. Thus it can be used in the areas of nonlinearities, lasing, modulators, and biosensors^4^,^5^. Large amount of researches have been devoted to generating the Fano resonances and studied the related sensing properties in various plasmonic structures^6^,^7^,^8^,^9^,^10^, such as nanoparticle clusters^6^, metamaterials^7^, and non-concentric cavity^8^ in the past several years. As another special property of the Fano resonance, the light speed in a Fano system can be significantly slowed down due to the steep phase dispersion^11^,^12^, which can enhance the light intensity by reason of the pulse compression. This unique characteristic can improve the sensing due to the increased light-mater interaction^13^. Huang *et al*. reported an enhanced sensor in a plasmonic waveguide^14^, and Wen *et al*. also studied the sensing with slow light in a fiber Bragg grating^15^. Here for clarity, we consider the former sensing in^6^,^7^,^8^ as universal sensing. Thus the Fano system possesses both the universal and the slow light sensing. However, those structures were either too bulky, complicated, or lossy, which are adverse to the compact and integrated device research.

Recently, plasmonic metal-dielectric-metal (MDM) waveguide have attracted people’s tremendous attention due to its remarkable advantages of long propagation distance, easy fabrication and wide available frequency range^16^,^17^, it can also be used for sensor applications^18^,^19^,^20^,^21^. For example, Lu *et al*. studied the plasmonic sensor in a MDM waveguide coupling with stubs^19^. Zafar *et al*. reported the enhanced figure of merit in a Fano resonance-based plasmonic sensor^21^. Nevertheless, these works mainly focused on the sensing performance numerically, no further discussion on regulating the figure of merit (FOM) of sensor^19^. In order to find out the effective factors those relate to the FOM and achieve the optimization of sensing properties, we need to establish theoretical models. However, still little attention is paid to the theoretical research on tuning both the universal and the slow light sensing properties in cavity coupled MDM waveguide system.

In this paper, we show a nanoplasmonic sensor based on the Fano resonance in a MDM waveguide systematically, it can be applied for both the universal and the slow-light sensing. The sensor is accurately tunable and the Fano asymmetric factor *p* is crucial to adjust the FOM of sensor. The contradiction in the slow-light sensing is discussed in details and can be solved by tuning the quality factor of the superradiant mode and the Fano asymmetric factor *p*. The presented theoretical model and the pronounced features of this simple plasmonic sensor, such as the tunable sensing and convenient integration, have significant applications in integrated compact plasmonic devices.

### Structure and analytic theory

**Figure 1:**
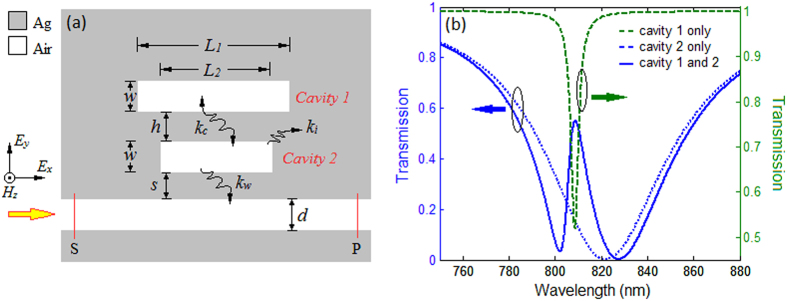
(**a**) The schematic of the plasmonic sensor. (**b**) Theoretical transmission spectra for single cavity coupled system and combined system. Parameters for the Fano type curve (blue solid) are: *ω*_0_ = 2.29 × 10^15^ rad/s, *Q*_*c*_ = 520, *Q*_*w*_ = 13.5, *Q*_*i*_ = 303, *C* = 0.998 and *ψ* = 3.9 rad. For the green dash curve are: *ω*_0_ = 2.33 × 10^15^ rad/s, *Q*_*w*_ = 753.6, *Q*_*c*_ = ∞. For the blue dash curve are: *ω*_0_ = 2.29 × 10^15^ rad/s, *Q*_*w*_ = 13.5, *Q*_*c*_ = ∞.

The schematic of the nanoplasmonic sensor is shown in Fig. 1(a). In this plasmonic system, the lower cavity with length *L*_2_ couples efficiently to the input wave in the bus waveguide thus be strongly excited, forming a wide continent spectrum. However, the upper cavity with length *L*_1_ couples weakly to the input wave and leads to a narrow discrete one. They can be analogously regarded as the superradiant mode and subradiant mode in forming the Fano resonance^19^. The superradiant cavity couples with the subradiant cavity through the evanescent field of SPs.

When the dielectric environment with refractive index *n* is filled in the entire system, the transmission coefficient at frequency *ω* can be derived by the coupled mode theory^22^,^23^ as


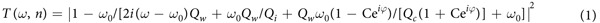


where *ω*_0_ is the resonant frequency of cavity 2, *φ* = 2*π*Re(*n*_*eff*_)*L*_1_/*λ* + *ψ* is the phase shift for a half roundtrip in cavity 1, *C* is the related attenuation coefficient, and *ψ* is the additional end-reflection phase shift in cavity 1. The effective refractive index *n*_*eff*_ in a MDM waveguide with width *w* can be obtained by solving the dispersion relation^24^. *Q*_*i*_, *Q*_*w*_, *Q*_*c*_ are the quality factors those represent the internal decay *k*_*i*_, the coupling decay to the waveguide *k*_*w*_ and to cavity 1 *k*_*c*_, respectively.

**Figure 2:**
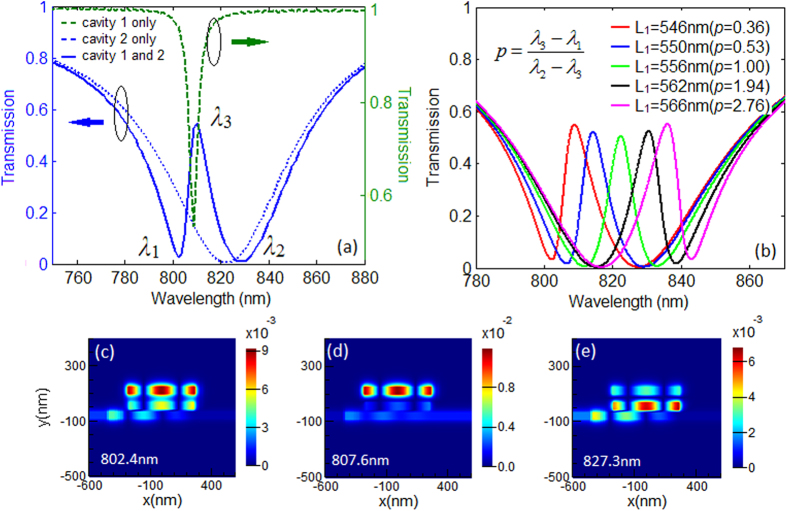
(**a**) Numerical transmission spectrum for structure with *L*_1_ = 546 nm, *L*_2_ = 530 nm, *w* = *d* = 50 nm, *s* = 20 nm and *h* = 60 nm. (**b**) Transmission spectra for different asymmetry degree factor. The magnetic field distributions at wavelength of dip *λ*_1_ (**c**), *λ*_2_ (**e**) and peak *λ*_3_ (**d**).

Figure 1(b) is the typical spectrum of a Fano resonance from equation (1). The wide blue dash curve is the transmission spectrum of the single lower cavity coupled system, while the narrow green dash curve is for the coupling waveguide system with a single upper cavity. Their coherent superposition leads to the asymmetric Fano lineshape, which is plotted as the solid blue curve. Figure 2(a) shows the 2D FDTD^25^ simulation results for structure with *L*_1_ = 546 nm, *L*_2_ = 530 nm, *w* = *d* = 50 nm, *s* = 20 nm and *h* = 60 nm. Two dips at 802.4 nm and 827.3 nm and the peak at 807.6 nm are marked as *λ*_1_, *λ*_2_ and *λ*_3_, respectively.

The optical features in Fig. 2(a) agree well with those in Fig. 1(b), which indicates that the physics mechanism of the Fano resonance can be best described by equation (1). For further investigation, here we define the asymmetry degree factor *p* of Fano line shape as *p* = (*λ*_3_ − *λ*_1_)/(*λ*_2_ − *λ*_3_). Figure 2(b) shows the transmission spectra for different *p*. Here *p* = 1 means a symmetric line shape as a special case, also known as the PIT effect^24^. As *p* increases from 0.36 to 2.76, the peak shifts to longer wavelength. Figure 2(c–e) are the related magnetic field distributions of transmission dips and peak wavelength in Fig. 2(a). We can see that at the Fano peak of 807.6 nm, the supperradiant mode is suppressed while excited for 802.4 and 827.3 nm.

Based on equation (1), when the system is surrounded by tested material with refractive index *n* + Δ*n*, the transmission coefficient then can be derived as


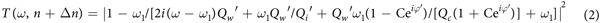


where *ω*_1_ = *ω*_0_/(*n* + Δ*n*), *Q*_*i*_′ and *Q*_*w*_′ are the resonance frequency and quality factors when the environment refractive index increases from *n* to *n* + Δ*n*, while *φ*′ is the half roundtrip phase shift in cavity 1. Those values are obtained due to the approximation that the line shape of the optical response spectrum nearly maintains the same, only leading to a red-shift^18^,^19^.

It’s known that there are two common ways in describing the Figure of merit (FOM) of a sensor^18^,^19^,^26^,^27^. One is related to the intensity variation at a certain wavelength^18^,^19^, and the other relates to the shift and width of resonance peak^26^,^27^. By considering that the detection used in some sensors, plasmonic biosensor, for example, is usually made by measuring the light intensity variation for one particular wavelength, here we give a precise analytical expression for FOM at frequency ω for the proposed nanoplasmonic sensor as


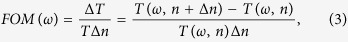


where *T*(*ω*, *n*) and *T*(*ω*, *n* + Δ*n*) have been obtained in equation (1) and (2), Δ*T* is the intensity variation at frequency *ω* caused by environment refractive index change Δ*n.*

## Discussions and Results

**Figure 3:**
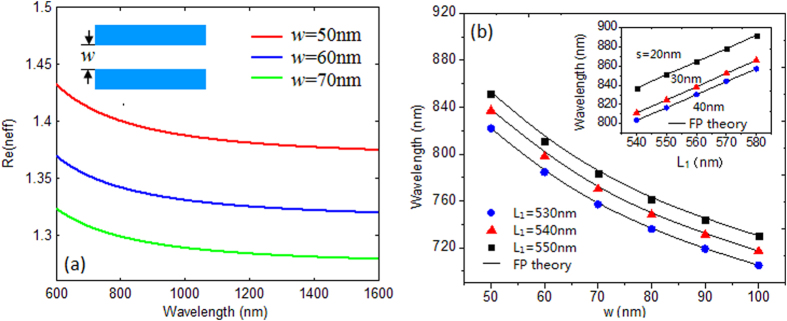
(**a**) Dispersion relation for MDM waveguides with various *w*. (**b**) Relation between resonance wavelength and cavity length *L*_1_ for different coupling distance.

According to the standing wave condition of a Fabry-Perot cavity^28^, the resonance wavelength can be adjusted by the length and the width of the cavity, which finally determine the asymmetry degree factor *p* of a Fano system. Figure 3(a) is the dispersion relation between the *n*_*eff*_ and the wavelength in a MDM waveguide with various *w*. We can see that the *n*_*eff*_ for narrower *w* is larger than that for the wider one. Figure 3(b) shows the resonance wavelength as functions of cavity length *L*_1_ with different coupling distance *s* for single cavity coupled MDM waveguide system. It’s found that the numerical wavelengths are consistent with the theoretical ones, which ensures the precise adjustment of Fano resonance.

**Figure 4:**
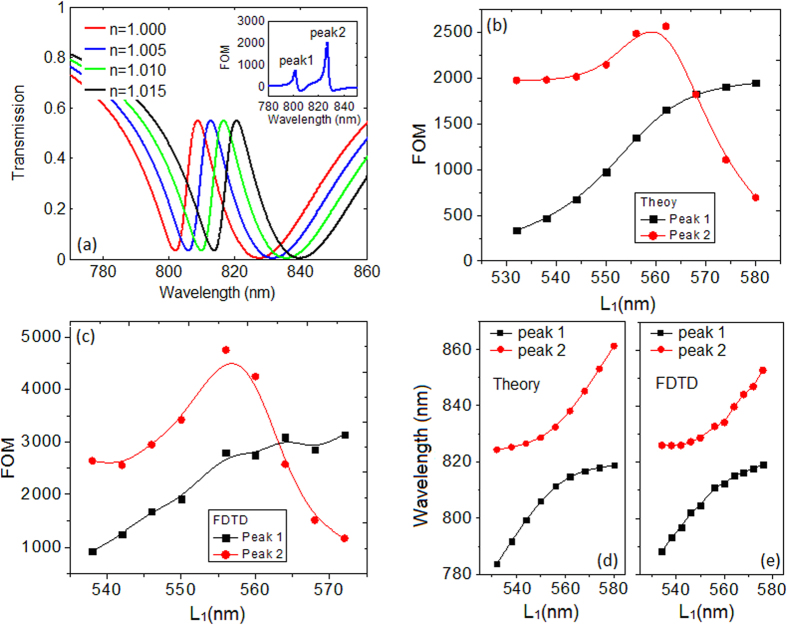
(**a**) The sensing response of the Fano-based nanosensor for varied dielectric environment. The inset shows the related FOM variation. (**b**) Theoretical and (**c**) numerical FOM as functions of *L*_1_. (**d**) Theoretical and (**e**) numerical wavelength locations for FOM maximums as functions of *L*_1_.

Since the Fano line shape can be precisely controlled, we now discuss the sensing performance of the proposed nanosensor. Here the parameters are those used in Fig. 2(a). In the following discussion, we only modify *L*_1_ while the others maintain the same. A typical sensing response can be found when the refractive index *n* of the environment increases from 1.000 to 1.015, shown as Fig. 4(a). By using equation (3), we get a FOM of ~2040 for dip 2 at 827.3 nm and 760 for dip 1 at 802.4 nm, which are much higher than that in^18^,^19^,^29^, and a sensitivity of 780 nm/RIU is also achieved. Those features can be beneficial for the sensing applications. The Fano spectrum in Fig. 4(a) is with an asymmetric factor *p* < 1, what will happen to the FOM if *p* gets enlarged? We first display the theoretical relations between the FOM and *L*_1_, shown in Fig. 4(b). It can be seen that as *L*_1_ increases from 532 nm to 580 nm, indicating an enlarging *p*, the FOM for dip 1 increases from 340 to 1948 accordingly. This is because the initial dip transmission *T* becomes smaller when *L*_1_ increases, seen in Fig. 2(b). In fact, the Δ*T* for dip 1 is also decreased due to the variational asymmetric degree of the line shape. Nevertheless, the total FOM for dip 1 still increases. Thus we can infer that the FOM for dip 1 is much more sensitive to the initial *T* rather than to the Δ*T*. For the sensing performance of dip 2, the FOM shows a nonmonotonic change. The FOM starts from 1978 and reaches a maximum of 2569 when *L*_1_ = 562 nm. But then drops down as *L*_1_ keeps on increasing. This interesting phenomenon can be explained by *T* and Δ*T* from the FOM definition. As *L*_1_ increases, the Fano peak shifts to longer wavelength. That will make both the Δ*T* and the initial *T* at dip 2 increase. Thus there seems to be a competition between those two factors, and the numerator Δ*T* plays the dominant role in tuning the FOM at the beginning. As *L*_1_ keeps increasing, this competition reaches a balance at *L*_1_ = 562 nm, leading to a maximum FOM of 2569. When *L*_1_ gets larger, the situation reversed. The denominator *T* turns to play the key role in adjusting the FOM. We conclude that for adjusting the FOM at dip 2, the competition between Δ*T* and *T* determines the FOM. For small *L*_1_, the intensity variation Δ*T* plays the key role, while for large *L*_1_, the initial *T* takes charge of it. Another interesting finding is that there is a cross point when *L*_1_ = 568 nm, this means two equal sensing peaks can be obtained by suitably choosing the cavity length. Thus this simple nanosensor also has potential applications as a double functional sensor, which is extremely sensitive to more than one wavelength (or frequency). To support those theoretical analysis and prediction, we provide the corresponding numerical results by FDTD simulations. The numerical relation between the FOM and *L*_1_ is shown in Fig. 4(c), and a highest FOM of ~4800 is achieved for *L*_1_ = 556 nm. We find that the tendency and law revealed in the numerical results agree well with those in the theoretical ones, which means the adjustment and evolution of FOM can be appropriately depicted by equation (3). The only difference between these two groups of result is the magnitude. The overall numerical FOMs are larger than the theoretical ones due to the relatively lower initial intensity *T* from the simulation. The relation between the wavelength where the FOM reaches its maximum and cavity length *L*_1_ is also discussed theoretically and numerically, shown in Fig. 4(d,e), respectively. The consistency of these two figures reveals that the location of the sensing peaks can be accurately predicted, which is beneficial for the sensor design. Here the solid curves in Fig. 4(b–e) are the B-spline fit to the data points. As we know, for subwavelength plasmonic sensing application, the very small volume that can be interrogated is also quite important. In the above discussion, we mainly focus on the dielectric environment change of the whole structure, including both the bus waveguide and the cavities. We now investigate the situation that the dielectric change only happens to some of the components (waveguide and cavities) of the nanoplasmonic sensor. Here we still consider the sensor in Fig. 2(a), and the cases of changing dielectric in cavity1 only, cavity 2 only and the waveguide only are briefly discussed, respectively. The corresponding FOM for the left (right) sensing peak are obtained as 695(634), 541(1770) and 5(35) for the above three types, which are smaller than 1676(2946) for the regular case, observed in Fig. 4(c). It is worth to note that, the extremely low FOM for the third group results from the fact that the spectrum showing relatively low sensitivity to the dielectric change in the bus waveguide. In addition, we also study the refractive index change in more than one component, such as cavity 1 and 2, cavity 1 and the waveguide, cavity 2 and the waveguide, respectively. And the corresponding FOM for the left (right) sensing peak are obtained as 1826(3263), 587(520) and 453(1522). Here the FOM for the first situation of 1826(3263) is close to 1676(2946) for the regular case, and the slight improvement may attribute to the decreased loss in the bus waveguide. These findings indicate that the sensing property can also be regulated by properly choosing the area where the sensing is actually taking place.

**Figure 5:**
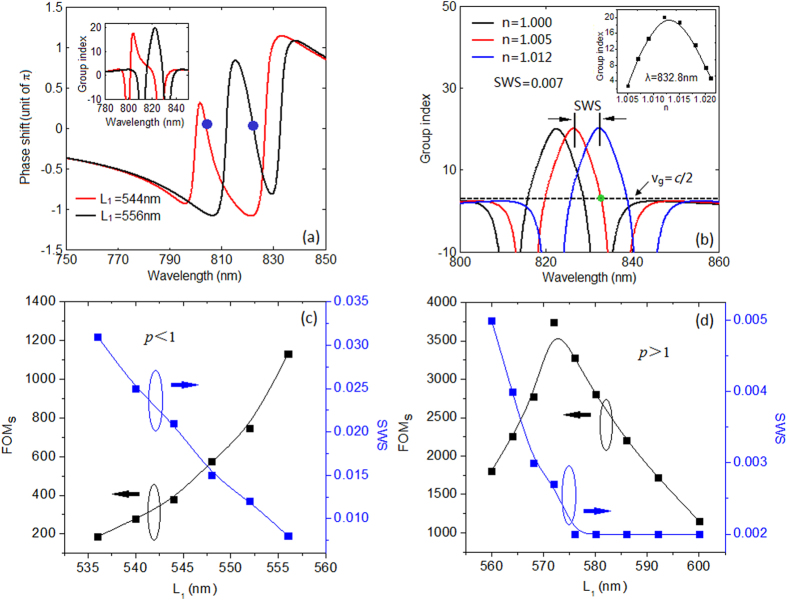
(**a**) The phase dispersion of the symmetric and the asymmetric systems. (**b**) The definition of the sensing width with slow-light. The FOM_S_ and SWS as functions of *L*_1_ for (**c**) *p* < 1 and (**d**) *p* > 1, respectively.

In the above section, we discuss the universal sensing based on Fano resonance in a cavity coupled MDM waveguide system, and find the way to accurately adjust the sensing property. Since the Fano resonance is the interference effect between the superadiant mode and the subradiant mode, the phase dispersion can be formed in a certain wavelength range and leads to a slow-light effect, shown in Fig. 5(a). The blue dots in the phase shift curve represent the locations of the maximum group index. The decrease of group velocity can enhance the light intensity by the pulse compression^13^, which will give rise to good sensing performance. Now we study the slow-light sensing in our Fano based sensor^15^. First we give the group index of this sensor system as *N*_*g*_ = *c*/*v*_*g*_ = (*c*/*H*) · *τ*_*g*_ = (*c*/*H*) · (*dθ*(*ω*)/*dω*)^30^, where *v*_*g*_ is the group velocity, *τ*_*g*_ is delay time, and the phase shift *θ(ω)* is the function of angular frequency *ω, H* = 1000 nm is the length of the plasmonic system. Figure 5(b) depicts the group index of a sensor with *L*_1_ = 556 nm for various *n*. For *n* = 1, dielectric as air, the group index reaches its maximum of ~20 at 822.4 nm for the transmission peak. Due to the fact that the maximum FOM of a sensor appears at the dip wavelength, we pay more attention to the group index variation at the two dips 811.8 and 832.8 nm. When *n* = 1.005, the group index of pulse at 832.8 nm reaches to 2, seen as the green crosspoint between the red curve and the black horizontal dash line, which means the group velocity can be halved and results in an enhanced intensity. When *n* = 1.012, the group index reaches the maximum. The inset of Fig. 5(b) is the group index for pulse at 832.8 nm with different *n*, which increases first and then drops.

While for wavelength of 811.8 nm, the group index nearly maintains the same when *n* increase. Briefly, the group index for wavelength at dip 1 is not sensitive to the environment, which means only a universal sensing here, just like the discussion in the former section. However, for wavelength at 832.8 nm, its group velocity is quite sensitive to the surrounding dielectric. If we consider *N*_*g*_ ≥ 2 as slow-light region, an optical pulse with specific wavelength will be slowed down in the sensor within a certain range of *n*. We define a sensing width with slow-light (SWS) as: 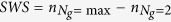 to characterize this certain range. 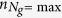 and 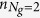 represent to the refractive index of dielectric environment for *N*_*g*_ reaching its maximum and 2, respectively.

For some particular wavelength, its pulse intensity can be enhanced during the sensing process. We also give this slow-light sensing an analytical expression based on the discussion in^31^. By utilizing the condition that *N*_*g*_ = *c*/*v*_*g*_, it can be further derived as


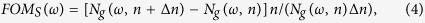


**Figure 6:**
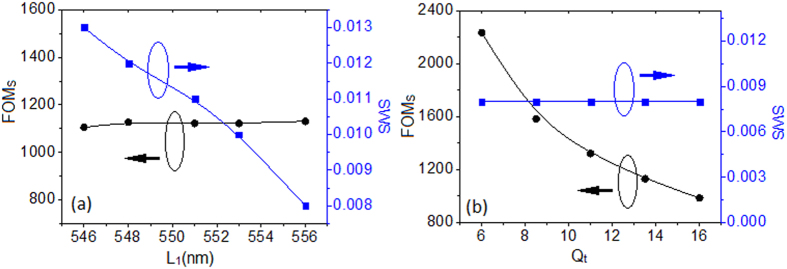
(**a**) The increased SWS without sacrificing the FOM_S_. (**b**) The increased FOM_S_ without sacrificing the SWS.

Here FOM_S_(*ω*) is the sensitivity of slow-light sensing, Δ*n* is chosen to be the SWS. Thus *N*_*g*_(*ω*, *n* + Δ*n*) is the maximum group index, and *N*_*g*_(*ω*, *n*) equals 2. We show the relation between the FOM_S_ and the SWS for different Fano asymmetric degree factor *p*. In Fig. 5(c), for *p* < 1, a tradeoff exists, and a maximum FOM_S_ of ~1131 is achieved when *L*_1_ = 556 nm, while the SWS reaches its highest value of 0.031 when *L*_1_ = 536 nm. This contradictory relation can be explained from equation (4). As *L*_1_ getting closer to 556 nm, the Fano line shape becomes more symmetric and the group index of the work wavelength increases and reaches its maximum of 20 when *L*_1_ = 556 nm. At the meantime, the SWS gets smaller since the slow-light band for dip 2 gets narrower. Thus for *L*_1_ = 556 nm, the highest group index while the narrowest SWS are obtained, which lead to a maximum FOM_S_. So the asymmetric degree factor *p* can also affect the slow light sensing, and we can get high slow-light sensitivity by making a symmetric line shape. Will the value of 1131 be the highest? And is the tendency reveals in Fig. 5(c) still suitable for larger *p*? The FOM_S_ and the SWS as functions for *L*_1_ > 556 nm (*p* > 1) are shown in Fig. 5(d). At the beginning, the FOM_S_ increases rather than decreases. Because both the *N*_*g*_ and the SWS become small, but the decreasing rate of SWS is faster than that of *N*_*g*_, thus leading to the increased slow-light sensitivity by equation (4). However, this increase trend stops and reaches a maximum of 3750 at *L*_1_ = 572 nm, this is probably a balance that the decreasing rate of the SWS is equal to that of the *N*_*g*_. After that, the SWS decreases slowly while the *N*_*g*_ decreases relatively fast, thus the FOM_S_ finally goes down. We can conclude that there is a tradeoff between the FOM_S_ and the SWS. Higher FOM_S_ usually results in lower SWS, which is also revealed in^23^. However, for real application, we need to make both of them large enough. Now we devote to make the SWS (FOM_S_) large enough without sacrificing the FOM_S_ (SWS). Fortunately, this contradiction can be overcome. Based on the qualitative analysis from equation (4), in order to make the SWS large enough without sacrificing the FOM_S_, this problem can be resolved by appropriately increase the *N*_*g*_ and the SWS of the Fano-based plasmonic sensor. These can be realized by adjusting the quality factor *Q*_*t*_ of the superradiant mode and the asymmetric degree *p* of the Fano line shape, which can increase the *N*_*g*_ and the SWS, respectively. Figure 6(a) shows the SWS and the FOM_S_ as functions of *L*_1_ for various quality factors *Q*_*t*_. Here the parameter *Q*_*t*_s are chosen as 6, 7, 8.5, 10 and 13.5 from left to right in the horizontal axis. As we properly decrease *Q*_*t*_ and *L*_1_, the SWS can be enlarged from 0.008 to 0.013 with a nearly unchanged FOM_S_ of 1127, which verifies the assumption in the former discussion. For the second problem, the solution can be simpler. We only need to decrease the quality factor *Q*_*t*_, which almost keeps the width of the slow-light band but increases the group index. By doing this we get an increasing FOM_S_ but nearly constant SWS. Here we deduce that the contradiction may also be resolved by approximately choosing the coupling distance *h* and consumption in system due to the fact that these two factors can also affect the SWS and the *N*_*g*_. The revealed contradiction relation between SWS and the FOM_S_ and the solutions may have potential applications in fundamental research and design of slow-light sensor.

## Conclusions

In conclusion, both the universal and the slow-light sensing can be achieved in a plasmonic MDM waveguide sensor based on Fano resonance. The consistency between the theoretical prediction and the numerical results in sensing property adjustment can be helpful in the sensor design and application. The Fano asymmetric factor *p* plays important role in adjusting both the universal and the slow-light sensing characteristic. The contradiction in slow-light sensing can be overcome by suitably tuning the loss of the superradiant mode and the Fano asymmetric factor *p*. The presented analysis and features of this simple plasmonic sensor can be useful in integrated compact plasmonic devices.

## Methods

The optical property of the silver nanostructure is approximated by the Drude model: *ε(ω)* = *ε*_*∞*_ − *ω*_*p*_^2^/*(ω*^2^ + *iωγ*_*p*_), with *ω*_*p*_ = 1.38 × 10^16^ s^−1^ is the bulk plasmon frequency, *ε*_*∞*_ = 3.7 and *γ*_*p*_ = 2.73 × 10^13^ s^−1^ represents the damping rate. The characteristic spectral responses of the structures are found by using the two-dimensional FDTD method with grid size Δ*x* = Δ*y* = 2.5 nm, S and P represent the incident and the detected plane, respectively. The calculation domain is surrounded by perfectly matched layer absorbing boundary. We choose FDTD solutions as our simulation software. And the simulation parameters have been given in our paper.

## Additional Information

**How to cite this article**: Zhan, S. *et al*. Tunable nanoplasmonic sensor based on the asymmetric degree of Fano resonance in MDM waveguide. *Sci. Rep.* **6**, 22428; doi: 10.1038/srep22428 (2016).
